# On-Site and Sensitive Pipeline Oxygen Detection Equipment Based on TDLAS

**DOI:** 10.3390/s25134027

**Published:** 2025-06-27

**Authors:** Yanfei Zhang, Kaiping Yuan, Zhaoan Yu, Yunhan Zhang, Xin Liu, Tieliang Lv

**Affiliations:** 1 State Key Laboratory of Integrated Chips and Systems, College of Integrated Circuits and Micro-Nano Electronics, Fudan University, Shanghai 200433, China; 20112020134@fudan.edu.cn (Y.Z.); kpyuan@fudan.edu.cn (K.Y.); 24112020117@m.fudan.edu.cn (Y.Z.); 21112020095@m.fudan.edu.cn (X.L.); 2 Frontier Institute of Chip and System Technology, Fudan University, Shanghai 200433, China; 3 Institute of Microelectronics, Chinese Academy of Sciences, Beijing 100029, China; yuzhaoan@ime.ac.cn; 4 School of microelectronics, Fudan University, Shanghai 200433, China

**Keywords:** tunable diode laser absorption spectroscopy, oxygen detection

## Abstract

The application of oxygen sensors based on Tunable Diode Laser Absorption Spectroscopy (TDLAS) in the industrial field has received extensive attention. However, most of the existing studies construct detection systems using discrete devices, making it difficult to apply them in the industrial field. In this work, through the optimization of the sensor circuit, the size of the core components of the sensor is reduced to 7.8 × 7.8 × 11.8 cm^3^, integrating the laser, photodetector, and system control circuit. A novel integrated optical path design is proposed for the optical mechanical structure, which enhances the structural integration and long-term optical path stability while reducing the system assembly complexity. The interlocking design of the laser-driven digital-to-analog converter (DAC) and photocurrent acquisition analog-to-digital converter (ADC) reduces the requirements of the harmonic signal extraction for the system hardware. By adopting a high-precision ADC and a high-resolution pulse-width modulation (PWM), the peak-to-peak value of the laser temperature control noise is reduced to 2 m°C, thereby reducing the detection noise of the sensor. This oxygen detection system has a minimum response time of 0.1 s. Under the condition of a 0.5 m detection optical path, the Allan variance shows that when the integration time is 5.6 s, the detection limit reaches 53.4 ppm, which is ahead of the detection accuracy of similar equipment under the very small system size.

## 1. Introduction

Oxygen plays a crucial role in the industrial production process, and its concentration directly affects the quality and safety of various industrial productions. Precise control of oxygen concentration can improve efficiency and reduce the generation of pollutants (e.g., CO, NO_x_, and SO_2_) in the industrial combustion process [1,2,3]. Oxygen concentration directly influences the composition and properties of products in the processes of metal smelting and ethylene-based ethylene oxide production [4,5,6]. Additionally, in the partial oxidation of natural gas for syngas production, oxygen levels are pivotal for maintaining a safe reaction environment [7,8,9]. Real-time detection of oxygen concentration in the gas inlet and outlet pipelines is of great significance during these industrial production processes as it enables real-time adjustment of oxygen concentration and process parameters. Tunable Diode Laser Absorption Spectroscopy (TDLAS) offers a powerful solution for gas concentration detection. Leveraging the unique absorption characteristics of gases to laser light, TDLAS measures oxygen levels by quantifying the attenuation of laser intensity as it passes through the target gas. Optical detectors capture the transmitted light intensity, enabling accurate concentration calculations. Renowned for its rapid response time, non-invasive operation, continuous real-time monitoring capabilities, and high measurement precision, TDLAS has demonstrated versatility in various oxygen detection applications [10,11,12,13,14].

Oxygen detection systems based on TDLAS have proven to possess significant application potential in engine combustion state monitoring [15,16], medicine vial airtightness inspection [17,18,19,20], and oxygen concentration monitoring in space stations [21]. However, the application of detecting oxygen concentration in industrial pipelines remains challenging. Current research predominantly relies on oxygen detection systems constructed from discrete components, such as DFB-2000 [10] and LDC-3742 [22] laser drivers, coupled with data acquisition cards for photoelectric signal collection [23]. When wavelength modulation spectroscopy (WMS) is employed, a sine signal must be superimposed on the laser driving current. Oxygen concentration is obtained by demodulating the high-frequency harmonic signal of the transmitted light intensity signal. Therefore, WMS necessitates a function generator to produce a sine modulation signal, enabling the wavelength modulation of the laser. Signal acquisition requires demodulating the harmonic signal using a lock-in amplifier or an oscilloscope, or calculating it using a field programmable gate array (FPGA) with high-speed signal processing capabilities [17,18,24]. Such discrete-based systems suffer from high costs and large form factors, rendering them unsuitable for on-site industrial pipeline measurements and impeding widespread adoption of oxygen sensors. Moreover, discrete devices increase system noise. These noises originate from the signal acquisition board, modulation drive module, signal demodulation module, and connecting cables, affecting the accuracy of the oxygen detection system. Therefore, a miniaturized, sensitive oxygen concentration detection device is needed to provide precise, on-site, real-time monitoring within industrial pipelines.

The work presents an on-site, sensitive oxygen detection system based on WMS, suitable for real-time, online monitoring of oxygen concentration in pipelines. The miniaturized system integrated a laser, a photodiode, and a system circuit, within a palm-sized form factor of 7.8 × 7.8 × 11.8 cm^3^. By integrating diffuse reflection principles with compact design in the system architecture, a novel optical path design method is proposed. This approach simplifies optical path assembly complexity, enhances long-term operational stability, and reduces sensor volume simultaneously. The system circuit enables directly output oxygen concentration, incorporating functions such as laser modulation and driving, photoelectric signal acquisition, harmonic signal demodulation and laser temperature control. The interlocking design of the laser-driven digital-to-analog converter (DAC) and photocurrent acquisition analog-to-digital converter (ADC) reduced the complexity of the harmonic signal extraction algorithm and circuit. By further optimizing the temperature control parameters and structural design of the laser system, we reduced the peak-to-peak value of laser temperature control noise from 10 to 2 m°C, quadrupling the stability of the second-harmonic signal. The system circuit used a microcontroller unit (MCU) to implement algorithms for laser modulation and driving, harmonic signal demodulation, and laser temperature control of the detection system, significantly enhancing the detection system’s integration level while reducing its volume. This oxygen detection system has a minimum response time of 0.1 s. Allan variance showed that the detection limit reached 53.4 ppm at the integration time of 5.6 s under a 0.5 m detection optical path. Small volume, fast response speed, and low detection limit endowed this system with great on-site application potential in industrial pipeline gas detection.

## 2. Principle and Methods

### 2.1. Principle

The principle of TDLAS for detecting gas concentration is based on the Lambert–Beer law. When a laser beam with an initial intensity of I0 and an initial frequency of υ passes through a certain uniform absorbing medium, intensity It(υ) of the transmitted light is defined by

(1) It(υ)=I0(υ)exp(−α(υ)CL)

where C represents gas concentration to be measured in the medium, which is the ratio of the number of gas molecules to be measured to the total number of gas molecules. L is the optical path length. α(υ) is the gas absorption coefficient, directly related to wavenumber υ of the laser. Meanwhile, it is complexly affected by broadening, gas temperature, and gas pressure. WMS is adopted in the work, requiring the modulation of laser wavenumber. The wavenumber modulation of the laser is achieved by modulating the laser current. Assuming that the modulation waveform of the laser is a cosine waveform, its driving current i(t) changes with time t as follows:

(2) i(t)=i0+Δicos(ωt)

where i0 is the driving current of the laser when center υ0 of absorption peak is locked; Δi is the current amplitude of the modulation current. Change υ(t) in laser frequency is defined by

**Figure 1:**
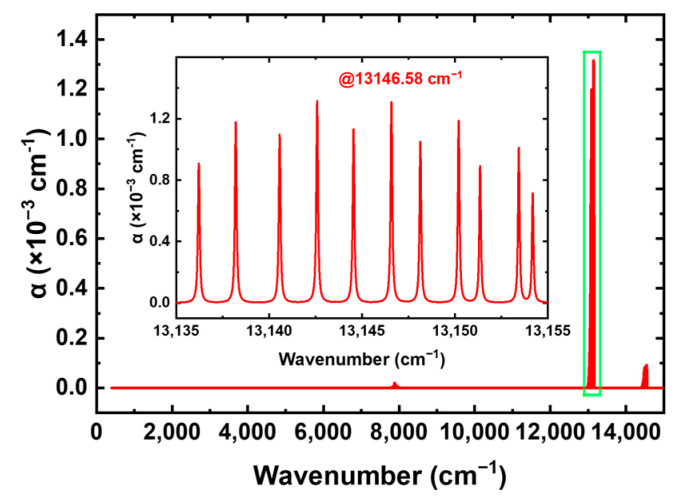
Oxygen absorption spectrum based on the HITRAN 2020 database [25].

(3) υ(t)=υ0+Δυcos(ωt+φ)

where υ0 is the center wavenumber of the absorption peak; Δυ is the amplitude of the laser modulation wavenumber; φ is the phase difference between laser current modulation and laser wavenumber modulation, which is the delay from the laser current modulation to the laser wavenumber modulation. As shown in Figure 1, the absorption peak of oxygen takes the relatively high absorption coefficient of 13,146.58 cm^−1^ as the detection center, corresponding to a laser wavelength of 760.654 nm. According to HITRAN database, CO_2_, CO, SO_x_, and NO_x_ exhibit no absorption in the 760 nm band. The absorption coefficient of water at 13,146.58 cm^−1^ is approximately 5 × 10^−8^ cm^−1^, and considering the maximum relative concentration of water in air is about 5%, its influence on oxygen detection can be neglected. Therefore, selecting this wavenumber for oxygen detection is the most appropriate choice. As the detection source, a Nanoplus 760.8 nm DFB (distributed feedback) laser is used.

A photodetector (PD) is required for detecting harmonic signals. The PD used in the work needed to detect near-infrared lasers with wavelengths around 760.8 nm; thus, the S5972 high-speed PD provided by Hamamatsu was adopted. This model operates normally at room temperature. Photosensitivity around 760 nm was approximately 0.52 A/W, and the temperature coefficient was lower than 0.1%, thus eliminating the need for a temperature control module. Upon light incidence, the PD generates a photocurrent, which is converted to a voltage via a custom-designed circuit. Fast Fourier transform processing of the photovoltaic data then extracts the harmonic signals critical for oxygen concentration analysis.

### 2.2. System Structure

**Figure 2:**
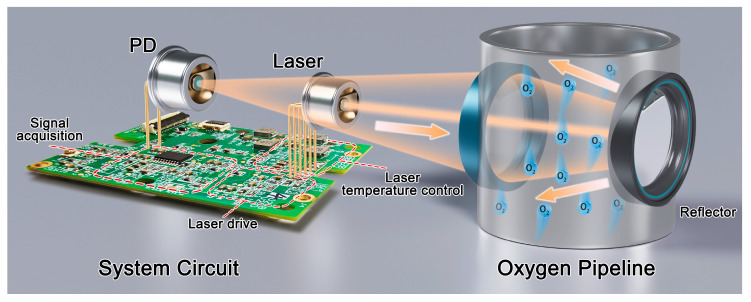
Pipeline oxygen detection system based on TDLAS.

The design of the system structure mainly takes into account the application of the device. As shown in Figure 2, the laser and the PD are mounted on one side of the pipeline to enable real-time oxygen concentration monitoring while simplifying the system architecture. The laser emits a 5–10 mW optical power beam that traverses the pipeline interior. The emitted light is collimated by a small lens with a 3.175 mm radius of curvature and 4.61 mm effective focal length, then passes through the pipeline gas where it undergoes absorption. Upon reaching the diffuse reflecting surface on the opposite pipeline wall, the laser beam is reflected back through the gas medium and returns to the emitter side. The PD, co-located with the laser on the same pipeline side, is positioned 55–65 mm (with adjustable spacing) from the exit window, detecting an optical power of approximately 7–20 μW.

The integrated design streamlines the optical path installation and calibration process, requiring only the core modules-laser and photodetector-to be calibrated, without involving other mechanical components. The diffuse reflection design ensures long-term optical path stability by minimizing the impact of mechanical misalignment on the optical path during prolonged operation. However, a single diffuse reflection configuration shortens the detection optical path, deteriorating the system’s detection limit. Therefore, it is essential to further optimize other system modules to improve the detection limit.

**Figure 3:**
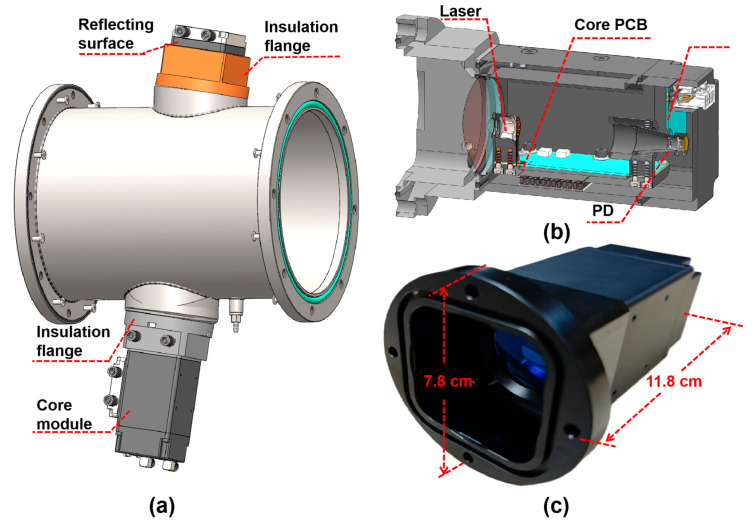
(**a**) 3D drawing of pipeline inspection design; (**b**) core module design section; (**c**) photograph of the core module.

The pipeline features a diameter of 150 mm, with the laser incident angle set at approximately 17°. The effective optical path length within the pipeline measures 313.86 mm, while the total optical path length extends to about 500 mm. For laser focusing, a lens is employed with a curvature radius of 31 mm and an effective focal length of 60 mm, demonstrating a measured transmittance of approximately 98.5%. The circuit board, laser and PD form the core module. As shown in Figure 3, the size of the core module is only 7.8 × 7.8 × 11.8 cm^3^. The compact structure design miniaturizes the sensor, which enhances the application potential of the sensor in the industrial field. This design allows for direct and rapid detection of oxygen concentration inside the pipeline, enabling online detection of oxygen concentration inside the pipeline.

### 2.3. System Circuit

Discrete systems typically utilize laser drivers, function generators, and oscilloscopes or lock-in amplifiers to control lasers, generate modulation signals, and detect harmonic signals, respectively. Discrete device-based detection systems suffer from high costs and large form factors, which is not conducive to large-scale application in the industrial field. Moreover, sensor systems constructed with discrete devices can generate extraneous noise, degrading system detection accuracy. Therefore, an integrated circuit should be designed to combine these functionalities and complete the miniaturized sensor system. This circuit portion should handle laser driving, signal acquisition, and laser temperature control. Optimizing the circuit design and sensor system can minimize the size and noise of the system.

**Figure 4:**
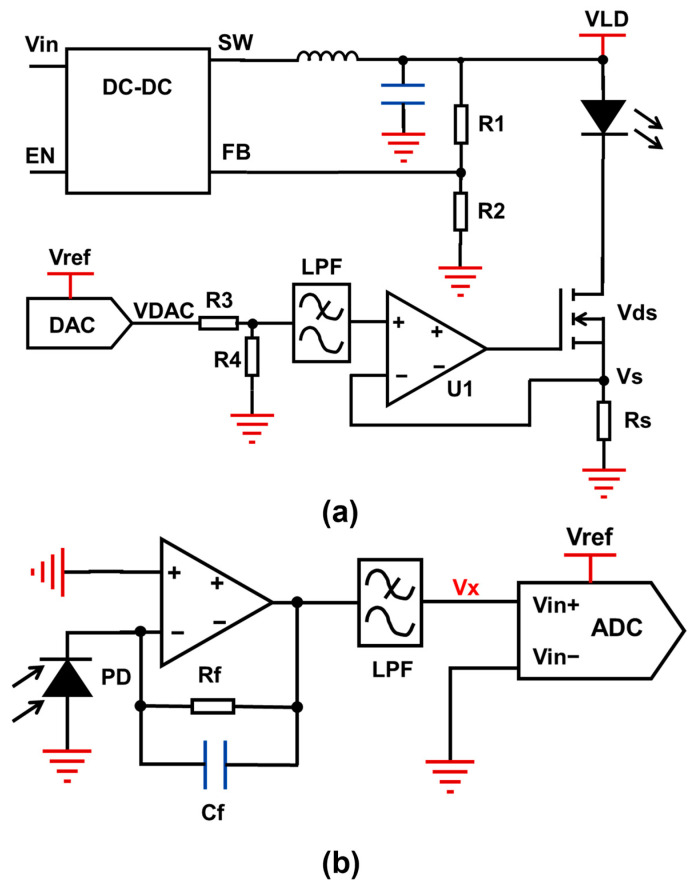
(**a**) Laser modulation drive circuit; (**b**) photocurrent signal amplification and acquisition circuit.

Figure 4a shows the laser driver circuit. The constant-current drive mainly relies on an N-metal-oxide-semiconductor (NMOS), constant-current resistor Rs, and operational amplifier U1 to form a negative-feedback constant-current control circuit. The digital-to-analog converter outputs a voltage (VDAC), which is divided by resistors R3 and R4, filtered by a low-pass filter (LPF), and then applied to the non-inverting input of the operational amplifier. Voltage-dividing resistors can reduce the power consumption of Rs and laser driver circuits. The upper end of constant-current resistance Rs is connected to the source terminal of the NMOS and then linked to the inverting input terminal of the operational amplifier, enabling negative feedback control where vs. equals the divided VDAC. The laser current depends on the ratio of vs. to Rs. For WMS, modulation signals (e.g., sine-wave signal) are superimposed on the original voltage of VDAC to modulate the laser current. The wavelength modulation frequency of the oxygen detection system is 1 kHz, and a 16-bit sine array is used to complete the wavelength modulation. The refresh frequency of the DAC is only 16 kHz. The used DAC model is LTC2641, and its −3 dB bandwidth is 1.3 MHz. For signals with a frequency lower than 100 kHz, the attenuation is negligible (≤0.1 dB). This type of DAC is fully capable of handling wavelength modulation tasks. Finally, the constant-current drive and wavelength modulation of the laser are achieved.

Conventional WMS systems typically require two function generators-one for constant-current control and another for modulation-to scan the gas absorption spectrum, necessitating an adder to combine signals. In contrast, this design uses a single DAC for both constant current and modulation drive, which reduces the complexity of the laser driver circuit and the volume of the detection system.

The photoelectric signal acquisition circuit is designed around the PD. Upon laser irradiation, the PD generates a photocurrent. A trans-impedance amplifier circuit is used to convert the photocurrent into photovoltage Vx. Output Vx is connected to the input interface of an ADC after passing through a low-pass filter and a subtractor. It is transmitted into the MCU through the serial peripheral interface (SPI) after being converted into a digital signal. The collected photoelectric signal undergoes demodulation to compute harmonic components for oxygen concentration detection, with the demodulation process involving Fourier transform of the photovoltage signal to extract harmonic signals.

Conventional discrete systems rely on lock-in amplifiers or oscilloscopes for harmonic detection, which suffer from bulky equipment that hinders system miniaturization. While integrated systems may use an FPGA for signal demodulation, this approach still challenges sensor miniaturization. To address this, the signal acquisition circuit is optimized by interlocking the laser-driving DAC and photovoltage-acquisition ADC, reducing both detection system volume and complexity.

Taking MCP3561 and LTC2642 as examples for ADC and DAC models, the two components share the same SPI interface in an interlocked configuration. The multifunctional ADC utilizes the MCLK, SCLK, SDI, SDO, nCS, and IRQ signals, with the IRQ signal connected to the external interrupt pin of the MCU. The DAC shares the SCLK and SDI of this SPI with the ADC. Chip-select signal nCS of the DAC is connected to the IRQ signal of the ADC through an inverter.

**Figure 5:**
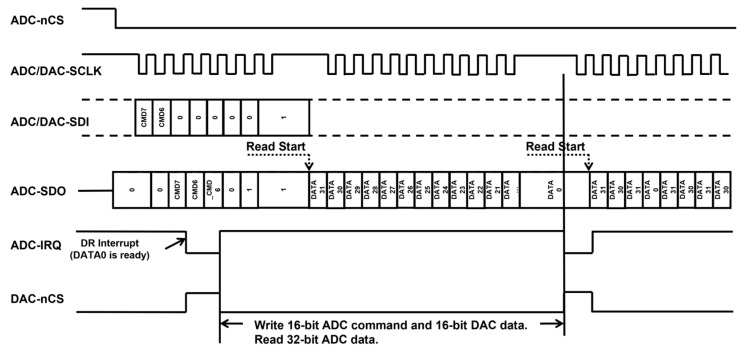
Logic timing diagram of ADC and DAC interlocking design.

The triggering timeline of the interlocking design is shown in Figure 5. When the ADC completes the data conversion, the IRQ signal level changes, triggering an external interrupt in the MCU. The MCU transmits a 16-bit command to the ADC to read the converted digital photovoltage signal during interrupt handling. Simultaneously, it continuously sends DAC data following this 16-bit command. Once the MCU finishes the reading of ADC data, the IRQ signal changes, and pulls up the nCS signal of the DAC through the inverter, prompting the DAC to convert the previously transmitted digital signal into an analog level. The first 16 bits of 32-bit data transmitted on the SDI each time correspond to the ADC read command; the last 16 bits are DAC data. The ADC is designed to recognize only the first digital signal transmitted on the SDI as a valid command, while the DAC is configured to recognize only the latest 16-bit digital signal received just before the nCS signal is pulled high. Consequently, any previously received digital signals will be overwritten and disregarded by the DAC. This circuit configuration and digital signal coordination enable complete ADC-DAC synchronization, ensuring stable and consistent phase difference between the two components.

A stable phase difference between the drive and sampling can simplify the system demodulation algorithm. The MCU can be directly used to perform the signal demodulation function. Equation (4) presents the demodulation algorithm. Compared with the common approach of using two separate SPIs to communicate with the ADC and DAC respectively, this scheme only requires a shared SPI to simultaneously achieve laser modulation driving and signal acquisition, simplifying system complexity. Furthermore, this method can further stabilize the phase difference of photoelectric signal acquisition. The stable phase difference can enhance the sensitivity of the detection system and reduce the harmonic signal demodulation noise caused by unstable clocks in the wavelength modulation method.

(4) Sn=1nn∑i=0nn−1V(i)(cos(n×i×2πnn)+jsin(n×i×2πnn))|Sn|=(Im(Sn))2+(Re(Sn))2

**Figure 6:**
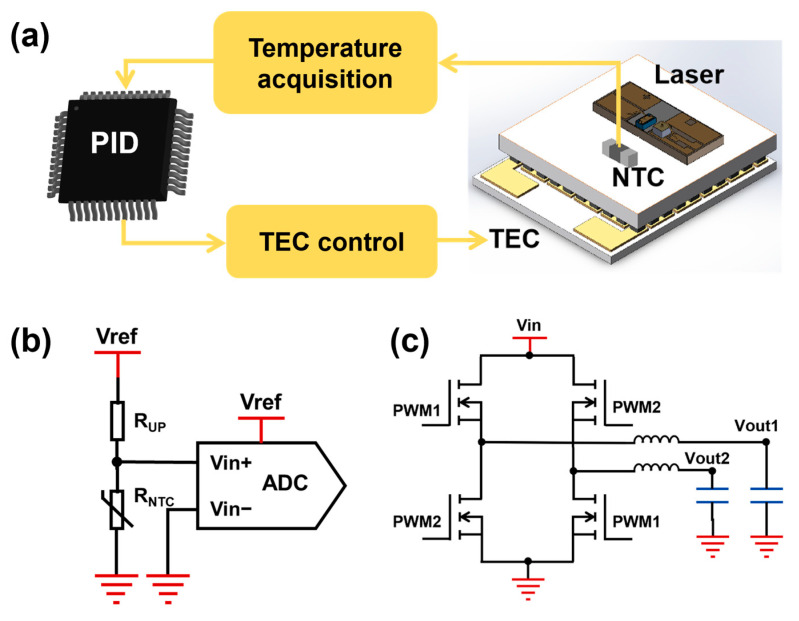
(**a**) Laser temperature control structure; (**b**) laser temperature acquisition circuit; (**c**) TEC drive circuit.

The laser temperature control circuit utilizes the Peltier effect of the TEC (thermoelectric cooler) to control the temperature of the laser. This circuit comprises two modules: a temperature information acquisition module and a TEC drive module (Figure 6). The temperature information acquisition module integrates a negative temperature coefficient (NTC) thermistor, a pull-up resistor, and an ADC. The resistance of the NTC changes with the temperature of the laser, which affects voltage Vin+, which is sampled by the ADC. Then the laser temperature is calculated inversely. Critically, the circuit’s reference voltage Vref shares the same source as the ADC’s reference, ensuring that any reference voltage noise is synchronized to suppress ripple and enhance temperature acquisition accuracy. The TEC drive module consists of an H-bridge chip, an inductor, and a capacitor. The switching of the H-bridge is achieved by inputting a pulse-width modulation (PWM) wave. Stable output voltages Vout1 and Vout2 are obtained after being filtered by the inductor and capacitor at the back end. The output voltage Vout correlates directly with the PWM duty cycle.

After setting the target laser temperature, real-time monitoring occurs by sampling the NTC resistance. A proportional-integral-derivative (PID) algorithm in the MCU calculates the required PWM duty cycle, which the H-bridge converts to TEC operating voltage, adjusting laser temperature for stable control. The PID algorithm executes every 0.1 s to ensure long-term stability. Compared with using external devices for temperature control, this temperature control circuit has a simple structure, which can reduce the size of the sensor. Besides, the temperature stability of the laser directly affects the detection accuracy of the sensor. This temperature control circuit has better temperature control stability, thereby enhancing sensor measurement precision.

**Figure 7:**
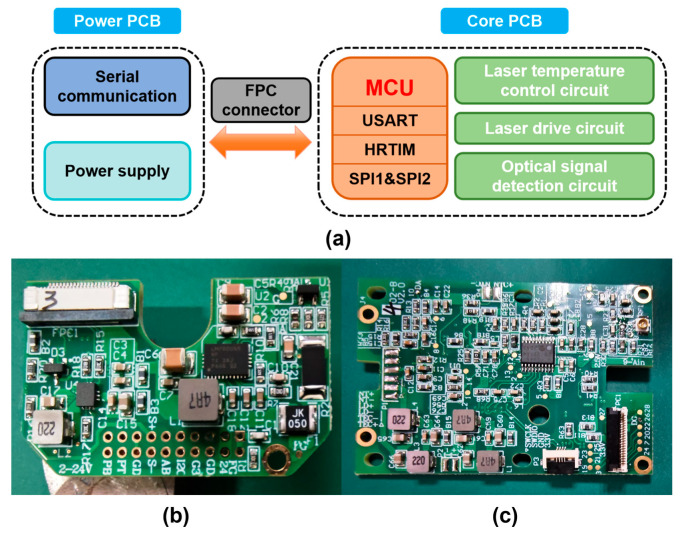
(**a**) Circuit board partition; (**b**) power board; (**c**) core board.

In addition to laser driving, photoelectric signal acquisition, and laser temperature control, the detection system circuit integrates modules such as serial communication and power supply. As shown in Figure 7, the circuit board is divided into a power board and a core board. The power board mainly includes the power supply and the communication module. The core board undertakes multiple functions, including laser driving, laser temperature control, and photoelectric detection modules. By strategically separating high-noise digital power and communication modules from the core detection circuitry, the design minimizes system detection noise and enhances gas detection accuracy. This board partitioning ensures that noise-generating components (e.g., switching power supplies, serial transceivers) do not interfere with low-level signal acquisition in the photodetector and laser control chains.

## 3. Results and Discussion

**Figure 8:**
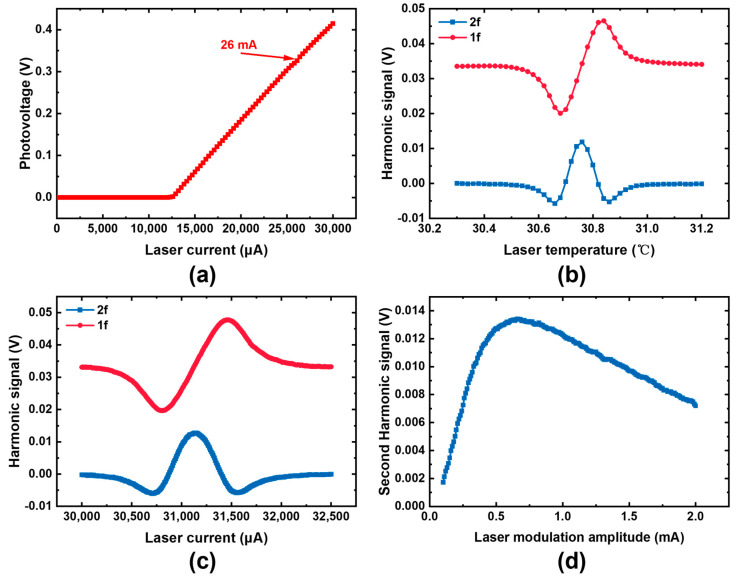
(**a**) Optical voltage under different laser currents; (**b**) harmonic signal under different laser temperatures; (**c**) harmonic signal under different laser currents; (**d**) second harmonic signal under different laser modulation amplitude.

The detection system, connected to the host computer via RS485 communication, enables multiple functions for calibrating the detection system. Figure 8 shows the calibration data of the laser tested by the detection system. The gas mixture used in the experimental tests is ambient air, primarily composed of 20.95% oxygen, 78.08% nitrogen, 0.03% carbon dioxide, and 0.93% inert gases.

First, a laser current-photovoltage scan is performed at 25 °C of the laser to determine its threshold current and the laser current value corresponding to the oxygen absorption peak. The threshold current of the laser is approximately 12.6 mA at 25 °C, and the laser current corresponding to the oxygen absorption peak is around 26 mA. The laser current is adjusted to 30 mA to provide a relatively large photovoltage. Next, a laser temperature scan is performed, identifying 30.5 °C as the optimal operating temperature. A laser current-harmonic signal scan is subsequently carried out, determining the laser’s operating current to be 31.140 mA. Finally, a laser modulation amplitude-harmonic signal scan is conducted, with the operating point selected at 0.66 mA-corresponding to the maximum value of the second-harmonic signal. Through this series of calibration processes, the laser’s operating parameters are determined as follows: an operating temperature of 30.5 °C, an operating current of 31.140 mA, and a modulation current amplitude of 0.66 mA.

After determining the sensor parameters, the oxygen detection absorption peak can be locked. Online detection of oxygen concentration is performed by detecting the ratio of the second-harmonic signal to the first-harmonic signal. The minimum detection time interval is 0.1 s, enabling rapid detection of oxygen concentration in the pipeline. However, further sensor system optimization is required to improve the accuracy of oxygen detection. The noise of this detection system mainly comes from the laser temperature control fluctuations.

The current tuning coefficient of this laser is approximately 0.241 cm^−1^/mA, and the temperature tuning coefficient is approximately 0.947 cm^−1^/°C. As described in typical literature, when the peak-to-peak value of the laser temperature control is 20 m°C [10,21,26,27], the corresponding standard deviation of the laser current control is 78.6 μA. Fitting results from current-scanned absorption peaks show that this leads to a 10.48% relative change in peak signal amplitude. Therefore, enhancing laser temperature control stability can significantly improve system detection accuracy and long-term operational reliability.

To enhance laser temperature control stability, the control system is partitioned into a temperature information acquisition module and a TEC voltage control module. A 24-bit precision ADC is used as the core chip for voltage acquisition in the temperature information acquisition module. The influence of the thermistor wire length on the temperature acquisition stability also needs to be considered in the actual layout and system design. Take a 10 kΩ color-coded resistor as an example: when its two-end leads are soldered to a printed circuit board (PCB), the ideal collected temperature at 25 °C often deviates in practice due to lead length affecting measurement stability.

**Figure 9:**
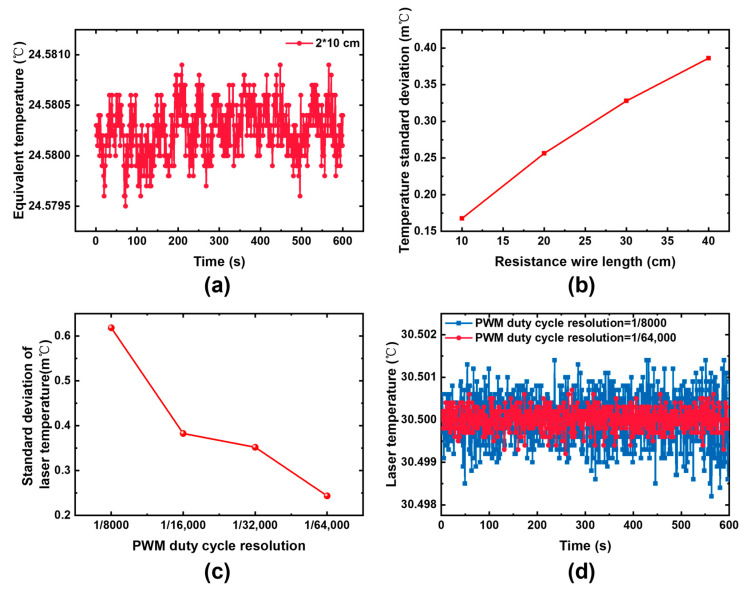
(**a**,**b**) Influence of resistance wire length on the stability of laser temperature acquisition; (**c**,**d**) influence of PWM resolution on laser temperature stability.

As shown in Figure 9, there is still noise in the equivalent temperature converted from the ADC sampling voltage after using 10 kΩ fixed-resistance resistors with different wire lengths. The standard deviation of the noise is affected by the resistor wiring length because longer resistor wiring is more susceptible to electromagnetic noise interference. Therefore, during PCB design, special measures should be taken to mitigate temperature and voltage acquisition noise, ensuring robust laser temperature control stability.

In addition to temperature acquisition, the laser temperature control module incorporates a TEC drive module via an H-bridge circuit. The accuracy of the TEC drive voltage directly affects the accuracy of TEC temperature control. The output voltage of the H-bridge is adjusted by modulating the input PWM duty cycle. Therefore, the temperature control precision directly influenced by the resolution of the PWM wave. A PWM with sufficient adjustment accuracy needs to be selected to improve the accuracy of TEC temperature control. The MCU model is the STM32G4x4 series. The internal clock of the MCU can reach up to 170 MHz, and the high-resolution clock function supports a maximum of 32× frequency multiplication. This enables the acquisition of a higher-resolution PWM wave as the laser temperature control source.

When PWMs with varying resolutions are used to control TEC drive voltage, the accuracy of laser temperature control increases with the increased PWM wave resolution. The peak-to-peak value of the laser temperature control noise is reduced to 2 m℃ by optimizing the temperature sampling circuit and the TEC drive circuit, which greatly enhances the operating stability of the sensor system.

Beyond steady-state temperature stability, the laser’s resilience to external thermal shocks should be addressed. Drastic external temperature changes can induce significant fluctuations in the laser’s high-stability temperature regime. Therefore, a second-order temperature control is integrated into the mechanical structure design. An additional TEC temperature control structure is added between the TEC and the heat sink to control temperature stability at the lower end of TEC1. When the external temperature changes rapidly, the temperature of the heat sink will change rapidly. The temperature change at the lower end of TEC1 is no longer significant after being adjusted by TEC2, which improves the laser’s resistance to external temperature shocks.

**Figure 10:**
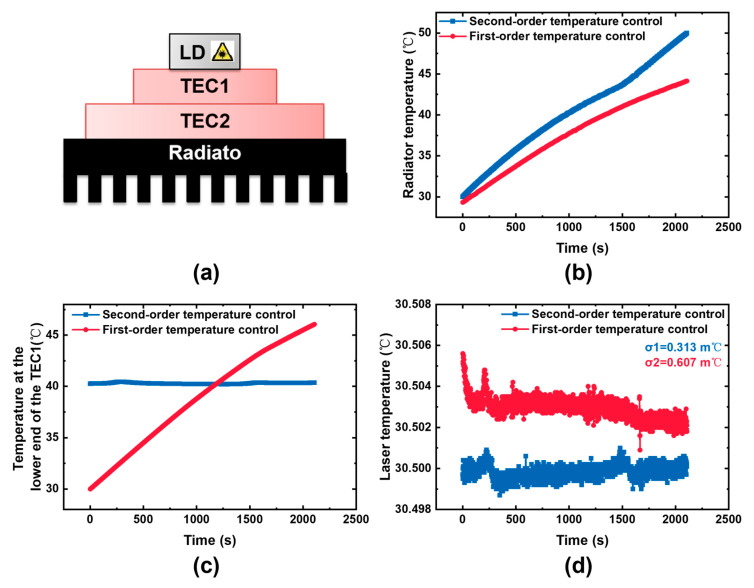
(**a**) Second-order temperature control structure; (**b**) effect of high-temperature shock on radiator temperature; (**c**) effect of high-temperature shock on the lower end of TEC1 temperature; (**d**) effect of high-temperature shock on laser temperature.

As shown in Figure 10, when exposed to rapid external temperature variations, such as the heat sink temperature rising from 30 to 50 °C in 30 min, the unoptimized first-order temperature-controlled TEC1 mirrors the heat sink’s upper-end temperature. This leads to severe temperature fluctuations, significantly degrading laser temperature control stability. A 3 m℃ increase in laser temperature directly shifts the oxygen absorption peak position, introducing approximately 0.5% detection signal error. Meanwhile, the temperature control standard deviation of the detection signal drops to 0.607 m℃, exacerbating system noise. By contrast, the implementation of second-order temperature control markedly mitigates these effects. The peak-to-peak value of the temperature change at the cold end of TEC1 is approximately 0.25 °C after adding the second-order temperature control, minimizing the impact of temperature shocks. The laser temperature is maintained at the target temperature of 30.5 °C, without significant temperature drift. The 10 min standard deviation of temperature control is 0.313 m℃, which is much lower than unoptimized 0.607 m℃. In summary, second-order temperature control enhances the system’s resistance to external temperature shocks. This improvement ensures stable laser temperature regulation in harsh environments and guarantees the accuracy and reliability of the detection system across complex operational scenarios.

**Figure 11:**
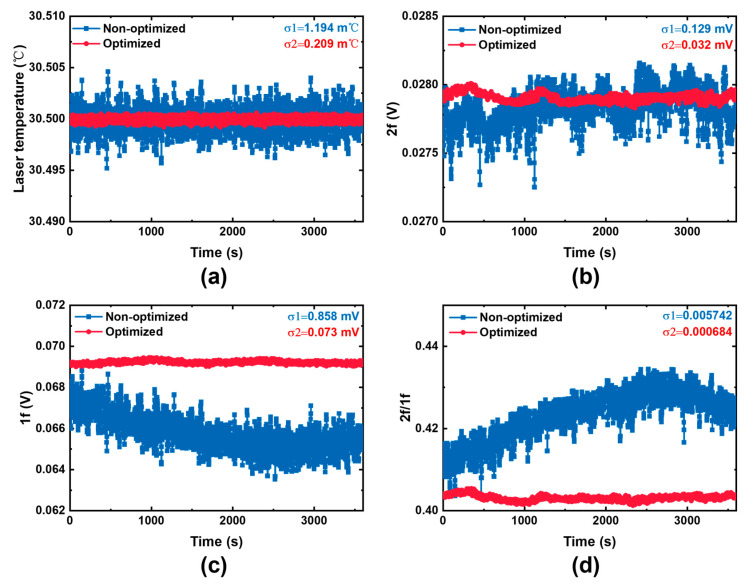
(**a**) Comparison of optimized laser temperature control; (**b**) influence of optimized laser temperature control on the second harmonic signal; (**c**) influence of optimized laser temperature control on the first harmonic signal; (**d**) influence of optimized laser temperature control on the ratio of the second harmonic to the first harmonic.

The signal stability of the detection system can be enhanced by optimizing the laser temperature acquisition circuit and the TEC drive circuit to improve the accuracy of laser temperature control. Compared with the unoptimized laser temperature control, one-hour stability of the optimized laser temperature control is improved from 1.194 to 0.209 m℃ (Figure 11). Simultaneously, the standard deviation of the second-harmonic stability of the detection system signal is reduced from 0.129 to 0.032 mV, while the standard deviation of the first–harmonic reduces substantially from 0.858 mV to 0.073 mV. Correspondingly, the ratio of the second-harmonic to the first-harmonic also experiences a significant decline, dropping from 0.005742 to 0.000684. The 5.7-fold enhancement in temperature control accuracy has profound implications for signal stability. It yields a fourfold increase in second-harmonic signal stability, an 11.7-fold boost in first-harmonic signal stability, and an 8.7-fold improvement in the stability of their ratio. Evidently, the heightened accuracy of laser temperature control within the detection system not only enhances the overall stability of the detection system but also effectively suppresses system noise, thereby contributing to more reliable and precise detection outcomes.

**Figure 12:**
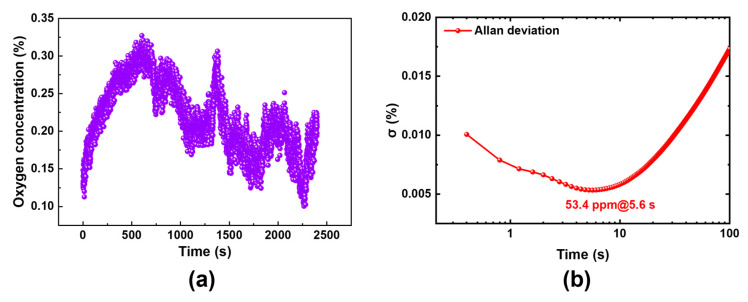
(**a**) Measurement of oxygen concentration in mixed air; (**b**) Allan deviation of the detection system.

Through comprehensive system circuit design and meticulous optimization of the laser temperature control system, this oxygen detection system achieves an exceptionally high detection limit. Unlike most conventional gas sensors that necessitate zero-concentration background gas for detecting the system’s limit, fluctuations in the TDLAS system primarily stem from internal components, rendering it independent of gas concentration. Therefore, it is feasible to use a gas of any known concentration to test the detection limit of the system. The pipeline gas with pure nitrogen is measured for 40 min at a detection frequency of 2.5 Hz. The detection limit reached a remarkably low value of only 53.4 ppm at an integration time of 5.6 s (Figure 12). As compared in Table 1, this performance far exceeds that of other oxygen detection systems based on wavelength modulation spectroscopy. The extremely low detection limit indicates the high reliability of this detection system and its broader application prospects.

## 4. Conclusions

Based on the WMS method, an on-site oxygen detection system centered around a laser and a PD was developed. The miniaturization of the sensor system was achieved by optimizing the sensor optical path and circuit. The integrated optical path design not only simplifies the assembly of the system’s optical circuitry but also reduces the sensor’s volume. Meanwhile, diffuse reflection enhances the long-term operational stability of the optical path. An ADC-DAC interlocking design was adopted to simplify the system circuit, enabling the MCU to extract the harmonic signal. A high-precision ADC and a high-resolution PWM were employed in the laser temperature control circuit, along with the digital PID algorithm in the MCU. The peak-to-peak value of the laser temperature control noise was reduced from 10 to 2 m°C, which resulted in a fourfold improvement in the stability of the second-harmonic signal. The laser, PD, control circuit, etc. were integrated into a core module. The size of this module is 7.8 × 7.8 × 11.8 cm^3^. The rapid on-site detection of oxygen concentration in industrial pipelines was achieved with a compact and miniaturized structure. The palm-sized sensor significantly enhanced its application potential in the industrial field. Allan variance indicated a 53.4 ppm detection limit for this oxygen detection system at a 0.5 m optical path and 5.6 s integration time, far exceeding the sensitivity of similar sensors. The extremely low detection limit and small volume demonstrate the high stability and ultra-high sensitivity of this detection system, which is expected to be applied in multiple industrial fields.

## Figures and Tables

**Table 1:** Comparison table of performance indicators of oxygen sensors.

Configuration	Optical Path Length	Detection Limit	Reference
WMS	0.296 m	610 ppm@181 s	[10]
WMS	n.a.	460 ppm@5.60 s	[20]
WMS	1.2 m	170 ppm@5 s	[28]
WMS	3.3 m	220 ppm@40 s	[29]
WMS	n.a.	583 ppm@100 s	[30]
WMS	0.5 m	53.4 ppm@5.6 s	This work

## Data Availability

The data presented in this study are available on request from the corresponding author.

## Abbreviations

The following abbreviations are used in this manuscript:

DAC	Digital-to-analog convertor
ADC	Analog-to-digital convertor
PWM	Pulse-width modulation
PID	Proportion-integration-differentiation
TDLAS	Tunable diode laser absorption spectroscopy
MCU	Microcontroller unit
DFB	Distributed feedback
PD	Photodetector
PCB	Printed circuit board
NMOS	N-metal-oxide-semiconductor
LPF	Low-pass filter
WMS	Wavelength modulation spectroscopy
FPGA	Field programmable gate array
NTC	Negative temperature coefficient
TEC	Thermoelectric cooler
